# Relationship Between Early Functional and Structural Brain Developments and Brain Injury in Preterm Infants

**DOI:** 10.1007/s12311-021-01232-z

**Published:** 2021-02-02

**Authors:** O. De Wel, S. Van Huffel, M. Lavanga, K. Jansen, A. Dereymaeker, J. Dudink, L. Gui, P. S. Hüppi, L. S. de Vries, G. Naulaers, M. J. N. L. Benders, M. L. Tataranno

**Affiliations:** 1 Department of Electrical Engineering (ESAT), STADIUS Center for Dynamical Systems, Signal Processing and Data Analytics, KU Leuven, Leuven, Belgium; 2 Department of Development and Regeneration, University Hospitals Leuven, Neonatal Intensive Care Unit & Child Neurology, KU Leuven, Leuven, Belgium; 3 Department of Development and Regeneration, University Hospitals Leuven, Neonatal Intensive Care Unit, KU Leuven, Leuven, Belgium; 4 Department of Neonatology, UMC Brain Center, Wilhelmina Children’s Hospital, Utrecht, The Netherlands; 5 Division of Development and Growth, Department of Pediatrics, Obstetrics & Gynaecology, University of Geneva, Geneva, Switzerland; 6 Department of Neonatology, University Medical Center Utrecht, KH.03.418.1, PO Box KE.04.123.1, Utrecht, The Netherlands

**Keywords:** EEG, Preterm infants, Neuromonitoring, Neuroimaging

## Abstract

**Background:**

Recent studies explored the relationship between early brain function and brain morphology, based on the hypothesis that increased brain activity can positively affect structural brain development and that excitatory neuronal activity stimulates myelination.

**Objective:**

To investigate the relationship between maturational features from early and serial aEEGs after premature birth and MRI metrics characterizing structural brain development and injury, measured around 30weeks postmenstrual age (PMA) and at term. Moreover, we aimed to verify whether previously developed maturational EEG features are related with PMA.

**Design/Methods:**

One hundred six extremely preterm infants received bedside aEEGs during the first 72h and weekly until week 5. 3T-MRIs were performed at 30weeks PMA and at term. Specific features were extracted to assess EEG maturation: (1) the spectral content, (2) the continuity [percentage of spontaneous activity transients (SAT%) and the interburst interval (IBI)], and (3) the complexity. Automatic MRI segmentation to assess volumes and MRI score was performed. The relationship between the maturational EEG features and MRI measures was investigated.

**Results:**

Both SAT% and EEG complexity were correlated with PMA. IBI was inversely associated with PMA. Complexity features had a positive correlation with the cerebellar size at 30weeks, while event-based measures were related to the cerebellar size at term. Cerebellar width, cortical grey matter, and total brain volume at term were inversely correlated with the relative power in the higher frequency bands.

**Conclusions:**

The continuity and complexity of the EEG steadily increase with increasing postnatal age. Increasing complexity and event-based features are associated with cerebellar size, a structure with enormous development during preterm life. Brain activity is important for later structural brain development.

**Supplementary Information:**

The online version contains supplementary material available at 10.1007/s12311-021-01232-z.

## Introduction

The immature brain of extremely preterm infants is prone to brain injury; therefore, monitoring and promoting optimal structural and functional brain development is key in the neonatal intensive care units (NICUs).

Electroencephalography (EEG) is an inexpensive and non-invasive tool widely used to perform long-term brain monitoring of preterm infants in the NICU. Previous research has focused on how the patterns and characteristics of the EEG evolve during preterm brain maturation. Studies have shown that maturational features extracted from serial EEG recordings can be used to construct a brain-age model and thereby assess the neonate’s brain function [1–4]. Moreover, maturational features can be useful for the prognosis of long-term neurodevelopmental outcome [5–8]. In addition, magnetic resonance imaging (MRI) is a valuable neuroimaging modality to detect preterm brain abnormalities, such as white matter injury and cerebellar hemorrhages, difficult to detect using cranial ultrasound [9, 10]. Sequential MRI allows longitudinal assessment of structural brain development, such as the integrity and growth of brain structures, and it is predictive of long-term outcome [11, 12]. Recent literature explored the relationship between early brain function assessed via aEEG in the first postnatal days and brain morphology evaluated at term using MRI in the preterm population. These studies hypothesized that increased brain activity can positively affect brain structural development and that excitatory neuronal activity stimulates myelination. In particular, neural activity was shown to be important for oligodendrocyte precursor cell proliferation, differentiation, and myelin biosynthesis [13]. These changes in myelin and oligodendrocyte precursors were also associated with an improvement in behavioral function, illustrating the importance of neuronal activity for myelin production in the developing mammalian brain [13]. In extremely preterm infants, early brain activity can be assessed by quantifying the occurrence of spontaneous activity transients (SATs). SATs are intermittent high-amplitude bursts of activity, typically seen in the preterm infants’ EEG [14–16]. They play a crucial role in human brain development, and their occurrence positively correlates to brain growth [17, 18]. The most common SAT events are the delta brushes appearing around 28 weeks postmenstrual age and disappearing around term [15, 19, 20]. On the other hand, longer periods of brain inactivity, described as “interburst intervals” (IBIs) were associated with poor outcome. A significant positive correlation between early brain activity, quantified using SAT rate and cerebellar and cortical grey matter growth between 30 and 40 weeks post menstrual age (PMA), was observed, while a negative association was shown with IBI [18]. Moreover, a higher SAT rate was accompanied by increased fractional anisotropy in the corpus callosum.

These results are supported by a recent study by Hüning et al. [10], exploring the relationship between aEEG and MRI features, and their power to predict neurodevelopmental outcome at 24 months [10]. By confirming the strong relationship between early brain activity and subsequent brain growth, they support the possibility that the combination of functional and structural brain parameters can aid in outcome prediction. However, none of these studies have investigated aEEG parameters beyond the first 3 days after birth. Moreover, the automated extraction of features from the aEEG is limited to the computation of SAT-related parameters, while many other maturational EEG features have been proven useful in the quantification of early brain function [1, 21]. At last, all previous studies were evaluated on a relatively small cohort of preterm infants.

The current study has two primary aims. The first objective is to perform a multicenter validation of state-of-the-art maturational EEG features. More specifically, we want to verify whether previously developed maturational EEG features also show a strong relationship with postmenstrual age on 2-channel aEEG measurements of a younger patient population acquired using different aEEG/EEG monitors.

Our secondary goal is to investigate the relationship between maturational features extracted from serial aEEG/EEG measurements and structural features derived from MRIs measured around 30 weeks PMA and at term equivalent age (TEA). In contrast with the previously described studies, the brain function is not only assessed during the perinatal adaptation period the first 72 h, but with serial aEEG/EEG recordings up to 5 weeks.

## Materials and Methods

### Study Design and Database

**Fig. 1:**
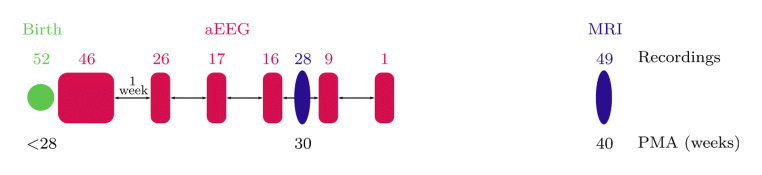
Visualization of the dataset used for the analysis. All babies are born before 28 weeks of gestation. **a** The 72h aEEG recording is measured immediately after birth, followed by weekly sequential recordings. Note that not all neonates are born at the same GA; hence, the first recording of one patient could for example be measured at the same PMA of the third recording of another patient. In 28 patients, an MRI is recorded as soon as possible after birth, around 30 weeks PMA. Additionally, an MRI is performed at TEA

The analysis was performed on aEEG and MRI recordings measured as part of the prospective NEOBRAIN study (ClinicalTrials.gov Identifier: NCT00544895; https://www.i-med.ac.at/neobrain/about/) between May 2008 and October 2010 at the Neonatal Intensive Care Unit of the Wilhelmina Children Hospital (Utrecht, The Netherlands). In total, 106 infants were enrolled in this study, all born extremely preterm, at a gestational age below 28 weeks. Patients with congenital disorders or syndromes were excluded from the study. Parental informed consent was signed, and the Utrecht medical ethical committee approved this observational retrospective study. A schematic overview of the different aspects of the dataset is given in Fig. 1.

### aEEG Acquisition

Bedside aEEG/EEG monitoring of each infant was started as soon as possible after birth and continued for the first 3 days after birth (72 h). In addition, sequential aEEG/EEG recordings with a duration between 2 and 4 h were performed each week for the first 4–5 consecutive weeks after birth, if patients were clinically stable.

The aEEG/EEGs were measured using four subcutaneous needle electrodes positioned over the parietal and frontal lobes (F3-P3 and F4-P4) according to the international 10–20 system. Additionally, a reference electrode measuring the impedance was placed on the central lobe. The aEEG/EEG was acquired at a sampling frequency of 256 Hz using either the BRM2 or BRM3 BrainZ monitor (BRM2/BRM3, BrainZ; Natus, Seattle, WA). The BRM2 and BRM3 monitor apply a high pass filter with a cutoff frequency at 2 Hz and 0.5 Hz, respectively.

### MRI Acquisition

MRI was performed at 30 weeks PMA and at TEA (PMA: 40-44 weeks) in all infants born below 28 weeks of gestation as standard of care at the Wilhelmina Children’s Hospital (WKZ). MRI at 30 weeks PMA was performed only if patients were respiratory and clinically stable. The TEA MRI was performed on a 3T MR system (Achieva, Philips Medical Systems, Best, the Netherlands) using a SENSE head coil. In order to reduce movement artifacts, the infants were positioned in a vacuum pillow. At 30 weeks PMA, MRI was performed using an MRI-compatible incubator (Dräger MR Incubator [Dräger, Lübeck, Germany] and later a Nomag IC 3.0 [LammersMedical Technology, Lübeck, Germany], with a dedicated neonatal head coil). Moreover, all infants received double-layer hearing protection using Minimuffs (Natus Medical Incorporated, San Carlos, CA) and Earmuffs (EM's 4 Kids, Brisbane, Australia) to reduce noise exposure.

The scanning protocol included T2-weighted imaging in the coronal plane (turbo spin echo, at 30 weeks: repetition time 10,085 ms; echo time 120ms; slice thickness 2 mm, in-plane spatial resolution 0.35 × 0.35 mm^2^; at TEA: repetition time 4847–6293 ms; echo time 120–150 ms; slice thickness 1.2 mm, in-plane spatial resolution 0.35 × 0.35 mm^2^, full brain coverage) for the calculation of brain volumes. A neonatologist or physician assistant, with experience in neonatal neurology, was present during the whole examination to check the patient’s condition and quality of the MRI images. Oxygen saturation, respiratory, and heart rate of the infants were continuously monitored. All infants were sedated using oral chloral hydrate 50–60 mg/kg at TEA, while no sedation was used at 30 weeks PMA.

### Data Preprocessing and EEG/MRI Features’ Extraction

When matching the EEG and MRI and checking the infants who had both good-quality EEG and MRI, only 49 infants remained in the analysis (see Fig. 1). An overview of all the features computed from on the one hand the EEG recordings and on the other hand the MR images is given in Table 1. An example of linear MRI measurements is shown in Supplemental figure 1.

**Table 1:** Overview of maturational features extracted from EEG recordings and developmental and injury measures extracted from MR images. The two rightmost columns indicate which features were extracted from the first (PMA 30 weeks) and/or the second (PMA 40 weeks) MRI recording

Modality		Feature		
Serial EEG		• Relative power in frequency subbands (delta, theta, alpha, beta)		
		• Spectral edge frequency 75%/90%		
		• SAT rate complete recording/5min		
		• Median IBI duration		
		• Complexity index		
		• Mean slope small scales [1–5]		
		• Maximum value MSE curve		
			30 weeks	40 weeks
Serial MRI	Development	• Cerebellar volume		✓
		• Cortical grey matter volume		✓
		• Volume intracranial cavity		✓
		• Cerebellar height (mid-sagittal)	✓	✓
		• Cerebellar width (mid-sagittal)	✓	✓
		• Cerebellar width (coronal)	✓	✓
	Injury	• Interhemispheric fissure	✓	✓
		• Total white matter injury score		✓
		• White matter classes		✓
		• Grey matter classes		✓
		• Ventricle left	✓	✓
		• Ventricle right	✓	✓

### EEG Preprocessing

Automated artifact detection revealed that many EEG recordings are heavily distorted by artifacts [22]. Therefore, the quality of the EEG recordings was visually evaluated in order to exclude severe abnormalities, artifacts, or caretaking events. During this manual data pre-selection, a representative 1-h EEG epoch was selected taking into account the sleep-wake cycling of the infant, including both active and quiet sleep when present.

From each of the three consecutive days of the first 72-h aEEG recording, the best-quality EEG epoch was selected. In four patients, the first EEG recording did not span the complete period of 5 days, and only two 1-h EEG epochs could be chosen. If the quality of the subsequent weekly recording allowed it, a 1-h epoch of clean EEG was visually selected. Thus, some poor-quality aEEG recordings were discarded from the database, and all further processing was performed on EEG epochs with a duration of 1 h. In total, 115 EEG recordings from 52 neonates were retained after visual assessment of the quality. In 46 of these neonates, the first recording of 72 h as soon as possible after birth was available, 26 recordings were measured approximately 1 week after birth, 17 were recorded around two weeks after birth, 16 recordings were measured in the subsequent week, and respectively 9 and 1 recording were registered at 4 and 5 weeks after birth. Fig. 1 illustrates how many EEG recordings were retained for further analyses at each time point.

Each of the manually preselected 1-h EEG epochs was then bandpass filtered to preserve the electrocortical activity of interest, while reducing the contribution of low- and high frequency artifacts. The bandwidth of a normal preterm EEG signal ranges from around 0.5 Hz up to 40 Hz. However, the BRM2 BrainZ monitor has a built-in high-pass filter with cutoff frequency 2 Hz. Therefore, all signals had to be filtered with a high-pass filter with 2 Hz cutoff frequency in order to compose a homogeneous database. Thus, all EEG time series are filtered between 2 and 40 Hz using a FIR high-pass filter with cutoff frequency 2 Hz followed by FIR low-pass filter with cutoff frequency 40 Hz. In addition, a notch filter at 50 Hz is used to remove any remaining power line interference.

### EEG Feature Extraction

After preprocessing, a set of features were extracted from the EEG time series. These features assess three characteristics of the changing EEG patterns during maturation: (1) the spectral content, (2) the continuity, and (3) the complexity of the EEG time series.

First of all, a set of commonly used spectral features, the relative power in the delta (*δ* 2–4 Hz), theta (*θ* 4–8 Hz), alpha (*α* 8–12 Hz), and beta (*β* 12–30 Hz) frequency band, was extracted. Moreover, the spectral edge frequency (SEF) with the edge at 75% and 90% was computed. These spectral features are derived from the power spectral density estimated using Welch’s method, computed in non-overlapping 25-s EEG segments for each channel.

**Fig. 2:**
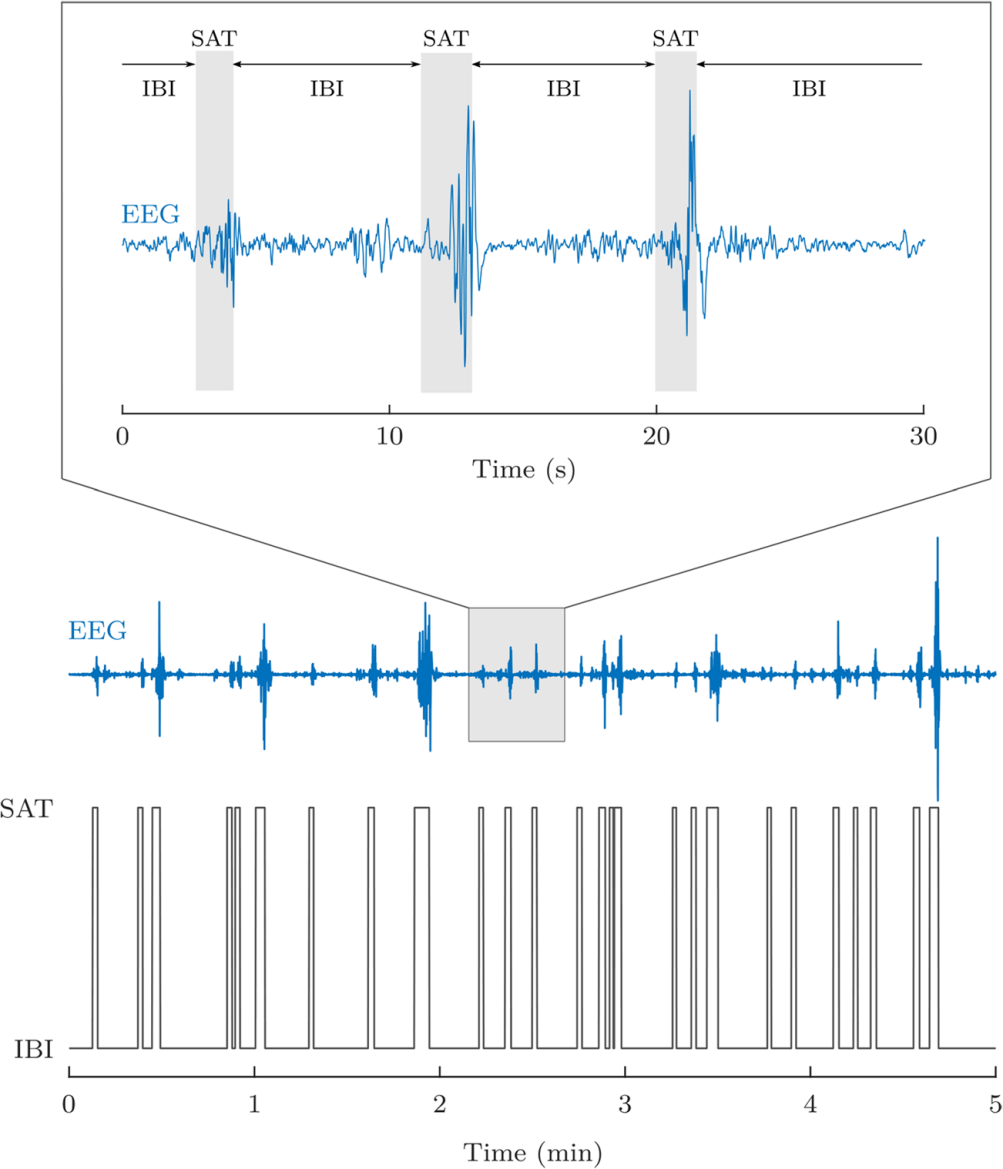
Illustration of the detection of spontaneous activity transient and interburst intervals in the preterm EEG. At the bottom, a 5-min EEG segment and the output of the NLEO-based SAT detection algorithm are presented. On top, a 30-s excerpt of the 5 min EEG segment is shown with labeled SAT events and IBI periods

The second type of EEG features was event-based EEG measures related to the continuity of the signal: the burst percentage or SAT% and the median interburst interval (IBI) duration. The SAT events are detected using the algorithm proposed by Palmu et al. [23]. This algorithm applies the nonlinear energy operator (NLEO) to the bandpass-filtered EEG time series. A threshold was then applied on the smoothed absolute value of the output of NLEO to identify the location of the SATs. Subsequently, the SAT percentage among the complete recording, the average SAT percentage in non-overlapping windows of 5 min, and the median IBI length among the complete EEG recording were computed. Fig. 2 illustrates the NLEO-based detection of SAT events and interburst intervals on a 5-min EEG segment.

At last, the complexity of each non-overlapping 100-s EEG segment is quantified using multiscale entropy [1, 24]. Multiscale entropy quantifies the complexity of a time series by assessing the sample entropy at multiple scales. The multiscale entropy curve, representing the sample entropy as a function of the scale *τ*, was then constructed per channel for scales ranging from 1 to 20. Three features were derived from this multiscale entropy curve: the complexity index which was computed as the area under the curve, the average slope in the small scales (*τ* 1–5), and the maximum value of the curve.

For all the above-mentioned maturational features, the grand average among all EEG segments within the 1-h selected clean EEG epoch was computed in order to obtain one feature value per recording (for each channel). In case of the first long recording, the average among the three clean 1-h EEG epochs was computed. Moreover, all features are averaged across the two channels. This averaging procedure relied on the fact that similar characteristics were expected in both hemispheres and will reduce the impact of short-duration distortions of the signal. As a result, one robust feature was obtained for each 2-channel aEEG recording.

### MRI Preprocessing

MR image quality was checked by two neonatologists with more than 20 years’ experience in neonatal neuroimaging (MB and LdV). The MRI was considered of appropriate quality to perform image postprocessing and compute brain volume measures in only 58 out of 106 infants.

### MRI Feature Extraction

In all images of appropriate quality, automatic MR image segmentation was performed with the atlas-free method of Gui et al. [25]. This yields segmentations of the following regions: cortical grey matter, unmyelinated white matter, myelinated white matter, subcortical grey matter, cerebrospinal fluid (CSF), cerebellum, and brain stem, obtaining the related volumes.

**Fig. 3:**
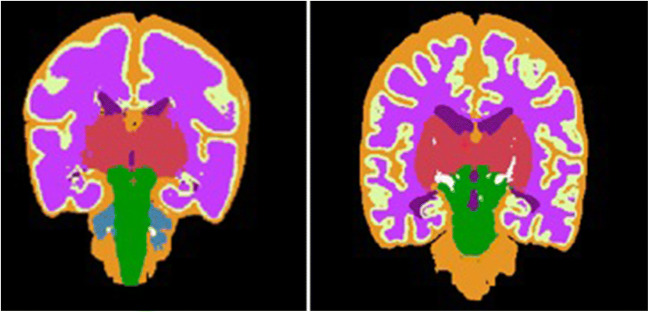
Examples of an automatically segmented MRI at **a** 30 weeks PMA and **b** 40 weeks PMA. Volumes are indicated according to the following color scheme: red: deep grey matter; pink: unmyelinated white matter; purple: ventricles; yellow: cortical grey matter; orange: cerebral spinal fluid; white: myelinated white matter; green: brain stem. blue: cerebellum

Total brain volume (TBV) was calculated as the sum of all brain region volumes, including the CSF. An example of a segmented MRI is presented in Figure 3. Because the MRI recordings were not all measured at the same postmenstrual age, brain volumes were adjusted for postmenstrual age at the time of the scan [26].

The brain volumes expected to be the most associated to early brain function (cerebellar volume, cortical grey matter volume, total brain volume) were retained for analysis as listed in Table 1.

In addition to volumetric measurements, the MRIs were also scored for brain injury by two experienced neonatologists according to Kidokoro et al. [27]. The Kidokoro MRI score assesses both brain injury and impaired brain growth. The MRI metrics derived from this assessment are the linear measurements of the cerebellar size in the coronal and mid-sagittal plane and all the brain injury metrics mentioned in Table 1.

### Brain Maturation Quantification Using EEG Features

A number of EEG features have been developed to track brain maturation in preterm infants. Many researchers have utilized characteristics related to the continuity of the EEG, because this is one of the main criteria in the visual assessment of neonatal EEG. However, more recently, also spectral and complexity features have been proposed. Since these features have not all been developed or validated on 2-channel EEG recordings of such a young preterm cohort, the first step in the analysis consists of investigating the relationship between each of the extracted maturational EEG features and the infant’s postmenstrual age (PMA) at the moment of the recording.

In total, 115 EEG recordings from 52 neonates measured at a PMA ranging from 24 weeks and 5 days to 31 weeks and 6 days were used for the analysis.

The Pearson correlation coefficient *ρ* was computed between each of the EEG metrics and the PMA. Subsequently, a regression model was built to investigate the relationship between these two variables. As multiple EEG recordings were measured from the same neonate, these recordings cannot be regarded as independent from each other. Therefore, a linear mixed-effects model accounting for the repeated measurements was implemented. A random intercept and slope were introduced to correct for inter-patient variability with the subject as grouping variable. For all statistical tests, the significance level was set at *p* < 0.05.

### Relationship Between Early Brain Function and Structure

#### Early EEG Metrics Versus Structural Brain Development and Brain Injury

After establishing which EEG features were suitable to track brain maturation in this group of preterm neonates, the relationship between the maturational EEG features and each of the MRI measures was investigated. In this first step, a similar approach to the existing literature [17] was taken. This means that the association between EEG features extracted from the first aEEG recording, during the first 72 h after birth, and MRI recordings performed at 30 and/or 40 weeks PMA was evaluated. This analysis could be performed on all 43 infants for which a clean first EEG recording and a high-quality MRI were available.

In addition, we wanted to examine whether neonates with increased early brain activity were characterized by increased brain growth between approximately 30 and 40 weeks PMA. This relationship could only be explored for the subset of 25 patients that underwent two serial MRI recordings and for the 3 developmental brain metrics that had been extracted from both MRI recordings. Therefore, only the change in size of the cerebellum in the mid-sagittal and coronal plane could be studied (see Table 1). The structural brain growth (increase/decrease per week) was computed as the difference between the feature at the second and first MRI recording divided by the number of weeks in between the recordings.

In the same way as described in the previous section, the relationship between the early brain function and structure was evaluated using a correlation and regression analysis.

The neonates included in this analysis were born at different gestational ages (GA: 24 weeks 5 days–27 weeks 6 days). Moreover, 26 out of 43 infants received morphine as sedation during mechanical ventilation. These variables might affect the found association. Therefore, the partial correlation between each of the EEG features and each of the MRI parameters, controlling for the gestational age and morphine administration, was computed.

Since only the first EEG measurement was used, all observations were independent, and there was no need to include random effects in the regression model. However, gestational age and morphine administration were included as additional independent variables in the model in order to control for confounding factors [28]. This resulted in a multiple regression model with an EEG feature, gestational age, and morphine administration as independent variables and the MRI metric as dependent variable. Thus, for each possible combination of EEG and MRI (growth) metrics, the partial correlation was computed and a multiple regression model was built.

#### Change in EEG Metrics Versus Structural Brain Development and Injury at Term Equivalent Age

In the second part of exploring the relationship between brain function and structure, the serial EEG recordings were exploited. As most of the neonates’ electrocortical activity was recorded at multiple time points, namely, immediately after birth and in subsequent weeks, a trajectory of the maturational EEG feature could be constructed per neonate. For the purpose of this analysis, all patients with multiple EEG recordings were selected, and the slope of these patient-specific maturation trajectories is estimated using a linear regression model. The hypothesis that was tested was that a rapidly increasing electrical early brain activity, expressed as a greater slope of the maturation trajectory, will be reflected in increased structural brain development on the MRI recording.

In total, 32 neonates had multiple EEG measurements, and an MRI recording at TEA, but for only 17 of these patients the early MRI was also available. Because of this small sample size, the relationship with features extracted from the early MRI measurements was not investigated.

For each of these 32 patients, a linear regression model was used to estimate the slope of the maturational feature, expressed as increase/decrease per week, as a function of PMA. The association between the change in early brain activity and each of the MRI metrics derived from the term recording was then explored by means of partial correlation and multiple linear regression accounting for the confounding factors GA and morphine administration.

## Results

Clinical characteristics of enrolled patients are shown in supplemental Table 1. Brain volumes of the subgroup of infants and MRI score following the method of Kidokoro et al. are shown in supplemental Table 2.

### Brain Maturation Quantification Using EEG Features

The results of the correlation analysis between each of the maturational EEG features and the PMA (left half of the table) and the results of regression analysis (right half of the table) are presented in Table 2.

**Table 2:** The results of the correlation and regression analysis performed to assess the relationship between the maturational EEG features and the postmenstrual age at the moment of the recording. The table reports the Pearson's correlation coefficient *ρ* and its *p* value. Moreover, the coefficient of determination *R*^2^, the regression coefficient *b*, and its 95% confidence interval (CI_lower_, CI_upper_) and *p* value are set out. Significant *p* values are in italics

EEG feature	Correlation		Regression		
	*ρ* (%)	*p* (*ρ*)	*R*^2^	*b* (CI_lower_, CI_upper_)	*p* (*b*)
Relative power *δ* band	−0.12	0.21	0.04	−3.29 (−9.2;2.62)	0.27
Relative power *θ* band	−0.02	0.82	0.05	−2.6 (−10.45;5.24)	0.51
Relative power *α* band	0.15	0.12	0.08	13.57 (−3.85;30.98)	0.13
Relative power *β* band	0.19	0.04	0.07	13.17 (0.52;25.83)	*0.04*
Spectral edge frequency 75%	0.13	0.15	0.05	0.29 (−0.11;0.69)	0.15
Spectral edge frequency 90%	0.26	0.00	0.07	0.34 (0.11;0.57)	*0.00*
SAT rate complete recording	0.52	0.00	0.27	0.08 (0.06;0.10)	*0.00*
Mean SAT rate 5 min	0.51	0.00	0.26	0.08 (0.05;0.10)	*0.00*
Median IBI duration	-0.29	0.00	0.20	−0.24 (−0.36; −0.12)	*0.00*
Complexity index	0.73	0.00	0.72	0.28 (0.24;0.33)	*0.00*
Mean slope small scales [1–5]	0.70	0.00	0.71	35.43 (29.59;41.26)	*0.00*
Maximum value MSE curve	0.72	0.00	0.71	4.52 (3.79;5.24)	*0.00*

Among the spectral features, the relative power in the beta band and the spectral edge frequency 90% are significantly correlated with the postmenstrual age of the neonate, *p* values 0.04 and 0.00 respectively (Table 2, left half). Moreover, the regression coefficients of these predictors are significantly different from 0 (13.17 and 0.34 respectively; Table 2, right half), meaning that regression analysis is also significant. However, the coefficient of determination is for both models equal to 0.07. This low R-squared value indicates that these models are not sufficiently reliable in predicting the age of the infant.

As expected, the SAT percentage computed in the complete recording or averaged within 5-min windows had a significant positive correlation with PMA (*p* value 0.00), while the median IBI duration decreased with increasing age (*p* value 0.00) (Table 2). These trends reflect the gradual increase of EEG continuity with maturation.

**Fig. 4:**
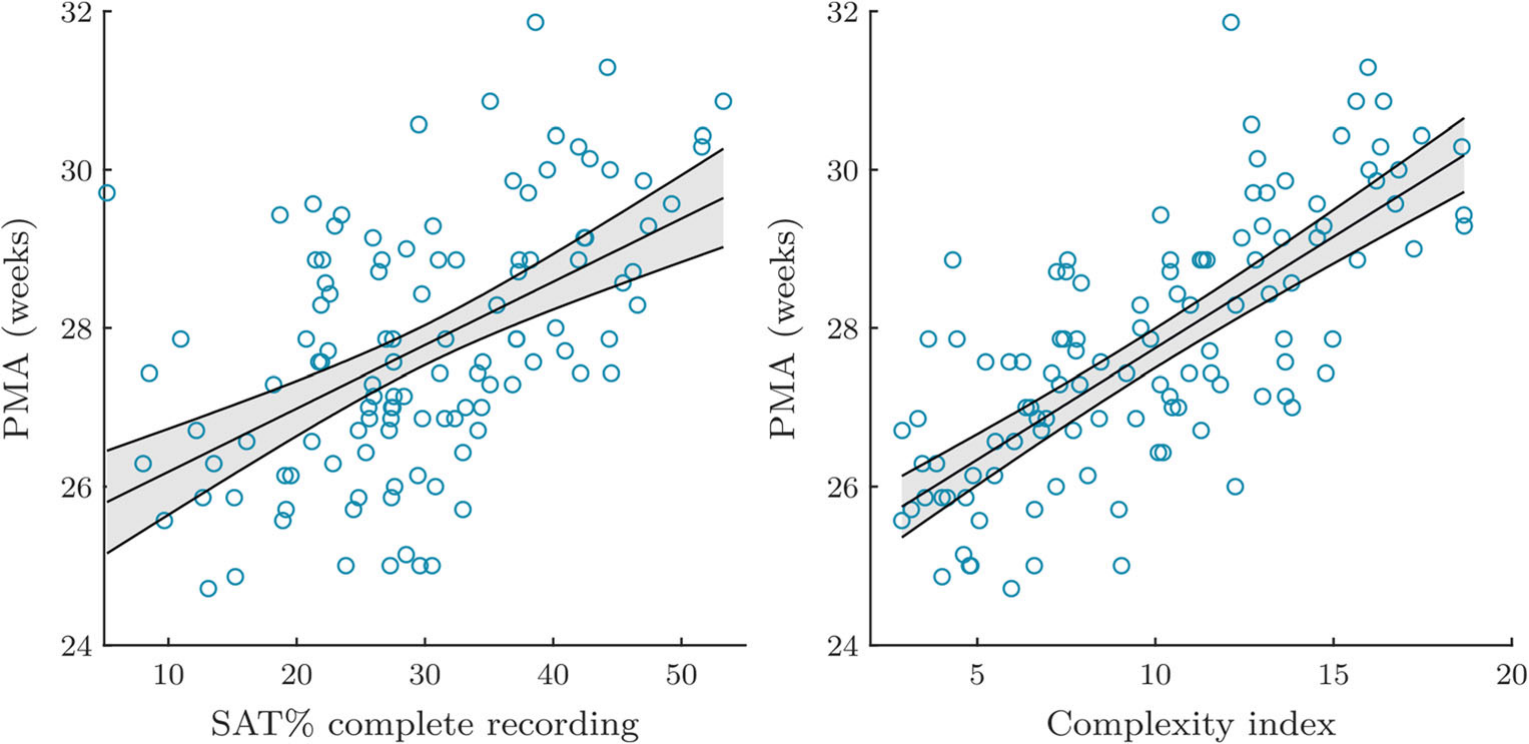
The fixed effects of the regression model fitting the relationship between SAT% and PMA are shown on the left. The regression model on the right shows the association between the complexity index and the PMA. Each blue circle represents a data point (corresponding to 1 EEG recording), while the black solid line shows the regression line. The 95% confidence bounds are indicated by the dashed lines, and the corresponding confidence interval is highlighted in light grey

The left graph in Fig. 4 shows the fixed effects of the regression model fitting the relationship between the SAT% and the PMA.

Finally, all maturational features related to the complexity of the EEG signal were strongly correlated with PMA (Table 2, last three rows left). Moreover, the fitted regression model has a significant positive slope and a coefficient of determination equal to 0.71 or 0.72 (Table 2, last three rows right). This strong positive correlation is also illustrated in Fig. 4 (right part), where the fixed effects of the regression between the complexity index and the PMA are visualized. It is apparent from Table 2 that the complexity features derived from the multiscale entropy curve were most associated with the age of the baby, followed by the SAT-related features.

### Relationship Between Early Brain Function and Structure

#### Early EEG Metrics Versus Structural Brain Development and Brain Injury

The results of the correlation and regression analysis for significantly correlated features are set out in Table 3. Only the EEG and MRI metrics which were significantly related will be presented.

**Table 3:** The relationship between early brain activity (recording during the first 72 h after birth) and features extracted from subsequent MRI recordings. The left column shows the MRI feature and the recording it was derived from (recording 1: around 30 weeks PMA, recording 2: around 40 weeks PMA). The next column lists the significantly associated EEG features, and the right part represents the results of the correlational and regression analysis. The correlation coefficient *ρ* and its corresponding *p* value *p*(*ρ*), the coefficient of determination *R*^*2*^, and the regression coefficient *b*, expressing the change in the MRI feature per unit increase of the EEG feature, and its *p* value *p*(*b*) are provided

MRI feature	Recording		EEG feature	Correlation		Regression		
	30 weeks	40 weeks		*ρ*	*p*(*ρ*)	*R*^2^	*b*(ci_lower_,ci_upper_)	*p*(*b*)
Cerebellar height (mid-sagittal)	✓		Complexity index	0.44	0.04	0.31	0.27 (0.01; 0.54)	0.04
	✓		Mean slope small scales	0.47	0.03	0.34	37.08 (4.35; 69.8)	0.03
Cerebellar width (mid-sagittal)		✓	Relative power alpha band	−0.45	0	0.23	−50.34 (−82.75; −17.92)	0.00
		✓	SAT%	0.55	0	0.33	0.13 (0.07; 0.19)	0.00
		✓	Mean SAT% 5min	0.55	0	0.33	0.13 (0.06; 0.19)	0.00
		✓	Median IBI duration	−0.42	0.01	0.2	−0.23 (−0.38; −0.07)	0.01
Growth cerebellar width (mid-sagittal)	✓	✓	SAT%	0.49	0.02	0.25	0.01 (0; 0.02)	0.02
	✓	✓	Mean SAT% 5min	0.49	0.02	0.25	0.01 (0; 0.02)	0.02
Cerebellar width (coronal)		✓	Relative power beta band	−0.56	0	0.43	−115.08 (−171.7; −58.47)	0.00
		✓	SEF90	−0.37	0.02	0.29	−1.27 (−2.31; −0.23)	0.02
Cerebellar volume		✓	SAT%	0.34	0.03	0.13	0.22 (0.02; 0.42)	0.03
		✓	Mean SAT% 5min	0.34	0.03	0.13	0.22 (0.02; 0.42)	0.03
		✓	Median IBI duration	−0.48	0	0.25	−0.73(−1.16; −0.3)	0.00
Cortical grey matter volume		✓	Relative power beta band	−0.37	0.02	0.15	−550.68 (−1004.25; −97.11)	0.02
Total brain volume		✓	Relative power beta band	−0.32	0.05	0.12	−1065.85 (−2131.15; −0.55)	0.05
Total white matter injury score		✓	Relative power beta band	0.31	0.05	0.12	32.8 (0.1; 65.49)	0.05
Ventricle right	✓		Complexity index	−0.43	0.05	0.22	−0.41 (−0.82; −0.01)	0.05
	✓		Mean slope small scales	−0.47	0.03	0.25	−57.42 (−107.8; −7.04)	0.03

Features related to the size of the cerebellum at either 30 weeks or 40 weeks PMA or the cerebellar growth were strongly associated with early brain activity (EEG recording in the first 72 h after birth). The complexity features were positively correlated with the linear measurement of the height of the cerebellum on the early MRI (30 weeks PMA), while the SAT-related features were more associated to the cerebellar linear measurements at TEA. The relative power in the higher frequency bands and the spectral edge frequency 90 were negatively related to the size of the cerebellum at TEA. In addition, the relative power in the beta band was also inversely correlated with the volume of the cortical grey matter and the total brain volume. The last three rows of Table 3 show the metrics for brain injury that were significantly correlated with early brain activity (EEG recording in the first 72 h after birth). The relative power of the EEG in the beta band is positively correlated with the total white matter injury score, while two complexity features were negatively correlated with the size of the right ventricle on the early MRI.

#### Change in EEG Metrics Versus Structural Brain Development and Injury at Term Equivalent Age

The results of the exploration of the association between the change in EEG metrics (weekly EEG) and the term MRI recording are presented in Table 4. The change in EEG metrics is closely related to the brain injury metrics, especially the size of the ventricles. Interestingly, a greater increase of the maximum value of the multiscale entropy curve is associated with a smaller left and right ventricle size. The slope of spectral features in the lower frequency ranges showed significant trends with the interhemispheric fissure (IHF), ventricular sizes, and the cerebellar volume. The change in the relative power in the delta and theta frequency band is respectively negatively and positively associated with the ventricular size.

**Table 4:** The relationship between the change in maturational EEG feature and the significantly correlated MRI metrics at TEA. The two leftmost columns show the MRI and EEG features of interest, respectively. The results of the correlation and regression analysis are presented at the right.

MRI feature	EEG feature	Correlation		Regression		
		*ρ*	*p*(*ρ*)	*R*^2^	*b*(ci_lower_,ci_upper_)	*p*(*b*)
Cerebellar volume	Relative power delta band	−0.4	0.04	0.18	−73.43(−142.34; −4.51)	0.04
IHF	Relative power theta band	0.39	0.03	0.2	21.92(1.75;42.1)	0.03
Ventricle left	Relative power delta band	−0.4	0.04	0.15	−25.84(−49.99; −1.7)	0.04
	Relative power theta band	0.45	0.01	0.2	41.6(9.8;73.41)	0.01
	Maximum value MSE curve	−0.4	0.04	0.14	−5.64(−11.13;-0.14)	0.04
Ventricle right	Relative power delta band	−0.4	0.01	0.21	−33.03(−58.92; −7.15)	0.01
	Relative power theta band	0.68	0	0.47	69.06(40.14;97.98)	0.00
	Maximum value MSE curve	−0.4	0.03	0.17	−6.65(−12.66; −0.65)	0.03

## Discussion

This study demonstrated that the discontinuity of the neonatal EEG decreases progressively during brain maturation. These results validate the EEG features previously proposed by our group to track early brain maturation in this patient group [1]. Furthermore, by investigating the relationship between the function and structure of the developing brain of preterm infants, this paper shows that increased brain function during the first weeks after birth reflects better later structural brain development and is influenced by brain injury, particularly by white matter injury.

Firstly, our results, assessing the direction and strength of the relationship between EEG features and PMA, evidence an increase of the burst percentage and a concomitant decrease of the median IBI duration in relation to PMA. The SAT% computed over the complete 1-h epoch of EEG and the average for consecutive 5-min segments were very similar, which is an indication of clean brain activity with little distortion due to artifacts. SATs represent the characteristic feature of the immature preterm brain activity [16, 29], and they disappear at TEA [29]. SATs are already detected at about 24 weeks, and in the early phase, they are mostly focal and concentrated over the sensory cortex [29], before wide spreading at later postnatal age. In contrast to earlier findings [21, 30], no maturational trend in the relative power of the delta, theta, alpha frequency band, or spectral edge frequency 75 could be observed. This result may be partly explained by the preprocessing of the EEG signals. Due to the different filter settings of the two BrainZ monitors (BRM2 and BRM3), all EEG time series had to be filtered with a high-pass filter with a cutoff frequency of 2 Hz, removing an important part of the delta frequency band. Consequently, all very low–frequency information of the EEG (0.5–2Hz), containing essential information, is lost. Only the relative power in the beta band (12–30 Hz) and spectral edge frequency 90 showed a significant positive correlation with PMA. However, the coefficient of determination of the corresponding regression models is only equal to 0.07, indicating a poor fit. The last set of EEG metrics assessed the complexity of the time series using features extracted from the multiscale entropy curve. These complexity features show the strongest relationship with PMA, with a correlation coefficient around 72% and a coefficient of determination of approximately 71%. These results are consistent with our previous findings [1] and with previous studies showing that EEG undergoes profound developmental changes during preterm life [15]. Hence, our results serve as multicenter validation on a younger patient group with 2-channel EEG recordings. As the continuity and complexity features were strongest related to the PMA of the neonate, we expect the evolution of these features to be the most related to the structural development of the brain and thus with MRI features.

The second main objective of the study was to determine whether early functional and structural developments are intertwined.

Two out of three complexity features were strongly positively correlated with the height of the cerebellum measured at 30 weeks PMA. Complexity features were also negatively correlated with ventricular size on the early MRI. Hence, more complex early (first 72 h after birth) EEG patterns seem to be related to the increased size of the cerebellum, while reduced EEG complexity is related to ventricular enlargement, probably indicating brain injury. Large ventricular size at term may be secondary to the reduction in the volume of the white matter [31–33]. These studies demonstrated also that the presence on larger ventricular size at term is an important and independent predictor of adverse cognitive and motor development at 4.5 years’ CA [31].

Cerebellum width at term was strongly associated with the decrease of EEG discontinuity. Moreover, increased burst percentage and shorter interburst intervals shortly after birth were associated with greater cerebellar volumes at TEA. At last, a higher SAT percentage is also positively correlated with subsequent increase of the cerebellar width. This finding is in agreement with the results of Tataranno et al. [18] who found that the number of SATs correlated with the cerebellar growth between 30 and 40 weeks PMA. SATs are the main hallmark of the preterm EEG, and previous studies have suggested their pivotal role in structural brain development [17, 29]. In particular, SATs are widely believed to be crucial for the establishment of early cortical circuits, neuronal differentiation, and synaptogenesis before external input via sensory systems begin to drive the activity-dependent brain development and plasticity [16, 34]. The cerebellar volume almost triples during the last trimester of gestation, making it one of the brain structures with the largest relative growth during this period, and as such, the developing cerebellum is highly vulnerable to multiple insults due to prematurity [35]. Moreover, there is strong evidence that injury to the cerebellum is associated with long-term cognitive, language, motor, and behavior impairment in extremely preterm infants [36–38]. The cerebellum receives and sends inputs to the cerebral cortex via a closed circuit with bidirectional feedbacks. Thus, the two structures (cerebellar and cerebral cortex) are strongly connected. The cerebellum receives inputs from many cerebral areas including also the prefrontal, posterior parietal, superior temporal, and limbic cortex. In addition, studies describe a changed contralateral cortical gray matter growth and functional disabilities in case of prematurity-related cerebellar injury [38, 39]. The present results strengthen the hypothesis that brain structural development seems dependent on and related to early brain activity. Thus, EEG can serve as an early biomarker for later brain development and injury.

Moreover, the EEG complexity features were related to the early MRI, while the EEG continuity features had significant associations with the MRI at TEA.

During maturation, the frequency of the preterm EEG increases slowly, which is captured by an increase of the spectral edge frequency and the relative power of the higher-frequency bands, while the relative power in the low-frequency bands decreases. This is in agreement with previous findings showing the same trend over PNA [16]. Furthermore, we observed that the cerebellar width, cortical grey matter volume, and total brain volume at term age are inversely correlated with the relative power in the higher-frequency bands (alpha and beta). Moreover, the relative power in the beta band is positively related to the total white matter injury score. The change in the relative power in the delta and theta band was also negatively associated to the ventricular size at TEA.

These unexpected findings possibly suggest that higher early relative power in the beta band might be not optimal for brain growth and might even be associated with white matter injury, whereas the high relative power in delta waves, appearing usually during sleep, might be needed for healthy brain growth and development. However, it is important to mention that other authors found that lower spectral EEG energies in the beta frequency range were associated with lower neurodevelopmental performance in healthy neonates [40]. Furthermore, preterm infants are known to experience an altered sleep architecture due to NICU environmental stressors and daily multiple painful procedures. The stressful and painful experiences in the NICU by preterm infants are associated with altered brain function and abnormal structural development [41].

Secondly, the increase/decrease of the EEG feature per week PMA were compared with the features of the term MRI. A smaller increase of the complexity during the first weeks after birth was related to larger left and right ventricles. Large lateral ventricles at 1 month in preterm infants were related to poorer motor development and to slower early language development at 2 years [42]. This demonstrates that EEG complexity might be suitable to detect brain injury. The key strength of the current study is that different aspects of the EEG time series are characterized. In contrast to earlier studies, we have not only looked at EEG features related to burst activity but also assessed the spectral content and complexity of the time series. Moreover, a broad range of MRI metrics quantifying either the development or the injury of the brain has been considered. Furthermore, the relation between the function and structure of the developing brain was extensively analyzed in a systematic way. Furthermore, both early and term MRI measures, and the growth between the two recordings, have been explored. In addition, the brain function was not only quantified during the first postnatal days. This is the first research where the maturational trend computed over serial EEG recordings is compared with structural brain development. However, a number of important limitations should be considered. Even though the current database consists of a larger patient group compared with previous research, there are some important drawbacks. Visual preselection of 1h EEG epochs was required because many of the EEG recordings were highly contaminated by artifacts. Due to this, a large proportion of each EEG recording was discarded, and the processing was not completely automated, making it infeasible for use in clinical practice. Many of the weekly recordings were not considered for analysis, as the poor quality did not allow the selection of 1 h of clean data. As a result of the small sample size of subsequent weekly recordings, these recordings could not be separately related to each of the MRI features, and the estimation of the slope of the maturation trajectory is less reliable. In addition, the fact that different BrainZ monitors were used to record the signals led to the loss of important spectral information from the delta frequency band. Besides, the study is limited by the lack of clinical annotations of sleep-wake cycling. Because of this, the analysis had to be performed for the complete EEG epoch, rather than on each sleep stage separately. Thus, for future studies, we aim to perform analysis on larger epochs (2–3h) so that our EEG segments could include at least one cycle for each sleep stage. At last, the number of early MRIs and the number of volumetric measures computed from these images are limited (e.g., volumes were only extracted at TEA). Future studies on the current topic are therefore recommended.

## Conclusion

In this study, the association between automatic early EEG analysis and MRI, providing information about the brain function and structure respectively, is investigated. First of all, we investigated which EEG features can be used to predict the PMA of the neonate, and in this way quantify brain maturation. Both SAT percentage and EEG complexity were significantly positively correlated with PMA, while the median IBI duration was inversely associated with age. This confirms that the EEG features previously proposed by our group are suitable to track early brain maturation and provide further support for the fact that with aging of the infant, the continuity and complexity of the preterm EEG steadily increases. A correlational analysis and multiple regression models were used to explore the relationship between early brain function and structural brain development. This analysis revealed that complexity features extracted from an EEG recording during the first 3 days after birth have a significant positive correlation with the cerebellar size around 30 weeks PMA, while the event-based measures are related to the cerebellar size at TEA. These results emphasize the importance of early brain activity for late brain development in extremely preterm infants and the possibility to use implemented automatic EEG algorithms for outcome prediction. Furthermore, it stresses the need for early neuromonitoring in these vulnerable newborns.

## Supplementary Information

ESM 1 (PNG 872 kb)

High resolution image (TIFF 2189 kb)

ESM 2 (DOC 56 kb)

## Data Availability

All anonymized data are openly accessible on request.
