# Supplementary figures and images for: Relationship Between Early Functional and Structural Brain Developments and Brain Injury in Preterm Infants
Source: Cerebellum. 2021 Feb 2;20(4):556–68. doi: 10.1007/s12311-021-01232-z (PMC8360868; doi:10.1007/s12311-021-01232-z)

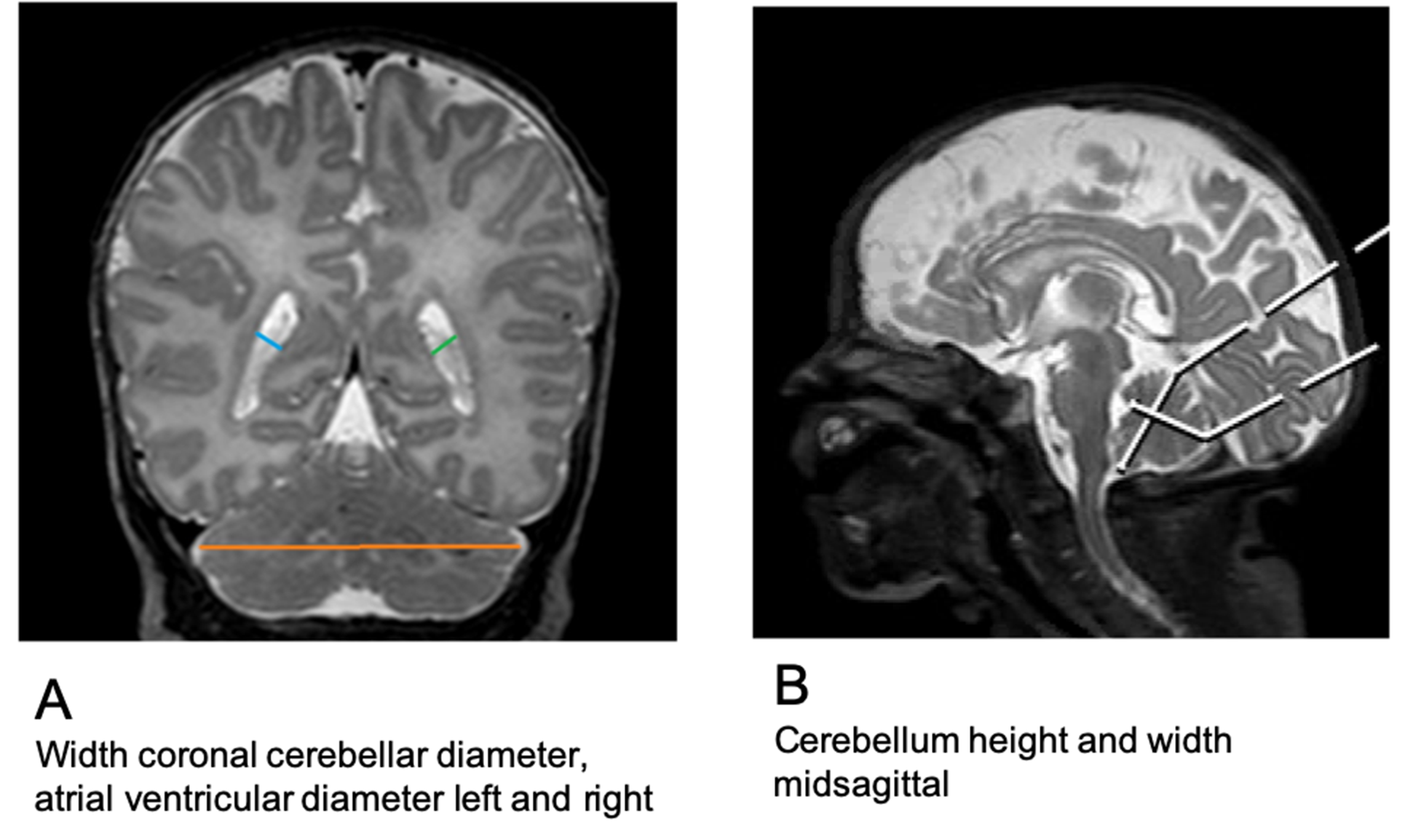

Supplement: Supplementary file 1 — (PNG 872 kb) [file 12311_2021_1232_Fig5_ESM.png]

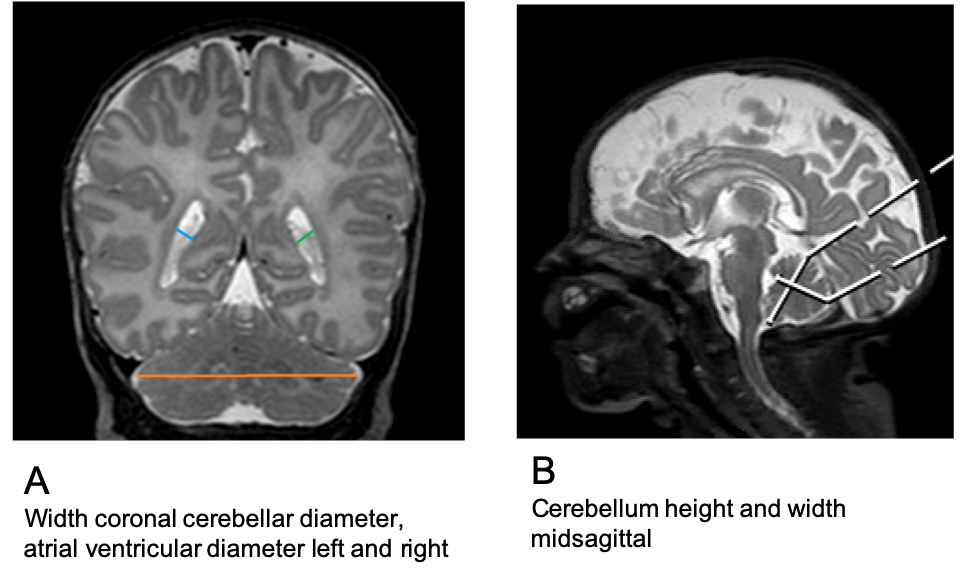

Supplement: Supplementary file 2 — High resolution image (TIFF 2189 kb) [file 12311_2021_1232_MOESM1_ESM.tiff]
