# Supplementary material for: Relationship Between Early Functional and Structural Brain Developments and Brain Injury in Preterm Infants
Source: Cerebellum. 2021 Feb 2;20(4):556–68. doi: 10.1007/s12311-021-01232-z (PMC8360868; doi:10.1007/s12311-021-01232-z)
Supplement: Supplementary file 3 — (DOC 56 kb) [file 12311_2021_1232_MOESM2_ESM.doc]

*Supplemental tables*

Supplemental table 1: Population characteristics. Abbreviations: GA=gestational age. BW=birth weight. IVH=intraventricular hemorrhage. PHVD= post-hemorrhagic ventricular dilatation. PDA=patent ductus arteriosus. NEC= necrotizing enterocolitis. BPD= bronchopulmonary dysplasia. PNA= postnatal age. SD=standard deviation. IR=interquartile range.

|  | | N=49 |
| --- | --- | --- |
| GA mean (SD) | | 26.4 (1.0) |
| Morphine yes n (%) | | 34 (69.4) |
| BW mean (SD) | | 901 (172) |
| Male gender n (%) | | 33 (67.3) |
| Cesarean section n (%) | | 23 (46.9) |
| Umbilical cord pH mean (SD) | | 7.28 (0.10) |
| Apgar score 1 min median (IR) | | 4 (2) |
| Apgar score 5 min median (IR) | | 7 (1) |
| IVH grade I-II n (%) | | 12 (24.5) |
| IVH grade III-PHVD n (%) | | 5 (10.2) |
| PDA n (%) | Medical treatment | 16 (32.7) |
|  | Surgery | 8 (16.3) |
| NEC n (%) | | 3 (6.1) |
| BPD n (%) | | 18 (36.7) |
| Sepsis n (%) | | 19 (38.8) |
| Cerebellum hemorrhage at 40 wks PNA n (%) | | 8 (16.2) |

# Supplemental table 2. MRI characteristics of the patients. WM=white matter, GM= grey matter, DGM= deep grey matter, CB= cerebellum, Global= global MRI score, ICV=intracranial volume.

| **MRI characteristics** |  |  | **N=49** |
| --- | --- | --- | --- |
| **Kidokoro MRI score** |  |  | 2 pt score missing |
|  | **WM** |  |  |
|  |  | normal n(%) | 28 (59,6) |
|  |  | mildly abnormal | 13 (27,7) |
|  |  | moderately abnormal | 5 (10,6) |
|  |  | severely abnormal | 1 (2,1) |
|  | **GM** | normal n(%) | 32 (68,1) |
|  |  | mildly abnormal | 10 (21,3) |
|  |  | moderately abnormal | 5 (10,6) |
|  |  | severely abnormal | 0 (0) |
|  | **DGM** | normal n(%) | 43 (91,5) |
|  |  | mildly abnormal | 3 (6,4) |
|  |  | moderately abnormal | 0 (0) |
|  |  | severely abnormal | 1 (2,1) |
|  | **CB** | normal n(%) | 31 (66) |
|  |  | mildly abnormal | 9 (19,1) |
|  |  | moderately abnormal | 6 (12,8) |
|  |  | severely abnormal | 1 (2,1) |
|  | **Global** | normal n(%) | 28 (59,6) |
|  |  | mildly abnormal | 14 (29,8) |
|  |  | moderately abnormal | 4 (8,5) |
|  |  | severely abnormal | 1 (2,1) |
| **Brain volumes (corrected for PNA at scan)** |  |  | **mean (SD)** |
|  |  | GM volume (cm3) | 171,46 (22,12) |
|  |  | cerebellum (cm3) | 22,61 (4,97) |
|  |  | ICV (cm3) | 441,54 (53,66) |
